# Transplant Oncology in Evolution: Emerging Roles for Liver Transplant Beyond Hepatocellular Carcinoma

**DOI:** 10.3390/cancers18060887

**Published:** 2026-03-10

**Authors:** Ahmed A. Abdelhakeem, Conor D. O’donnell, Dina Elantably, Oluwatayo Adeoye, Hani M. Babiker, Jason Starr, Liu Yang, Jordan D. Legout, Beau Toskich, Denise M. Harnois, Jeremy C. Jones, Kristopher P. Croome, Umair Majeed

**Affiliations:** 1 Department of Medicine, Division of Hematology-Oncology, Mayo Clinic Florida, Jacksonville, FL 32224, USA; abdelhakeem.ahmed@mayo.edu (A.A.A.); odonnell.conor@mayo.edu (C.D.O.); elantably.dina2@mayo.edu (D.E.); babiker.hani@mayo.edu (H.M.B.); starr.jason@mayo.edu (J.S.); jones.jeremy1@mayo.edu (J.C.J.); 2 Department of Medicine, Division of Gastroenterology and Hepatology, Mayo Clinic Florida, Jacksonville, FL 32224, USA; yang.liu@mayo.edu (L.Y.); harnois.denise@mayo.edu (D.M.H.); 3 Department of Radiology, Division of Interventional Radiology, Mayo Clinic Florida, Jacksonville, FL 32224, USAtoskich.beau@mayo.edu (B.T.); 4 Department of Transplant, Mayo Clinic Florida, Jacksonville, FL 32224, USA; croome.kristopher@mayo.edu

**Keywords:** liver transplantation, colorectal liver metastases, cholangiocarcinoma, perihilar, intrahepatic

## Abstract

Liver transplantation has emerged as a curative treatment option for selected patients with unresectable hepatic malignancies beyond hepatocellular carcinoma, marking a paradigm shift in transplant oncology. For colorectal cancer liver metastases (CRLM), prospective trials have demonstrated that highly selected patients achieve 5-year OS rates of 60–83%, with the Oslo score identifying optimal candidates for transplantation. Perihilar cholangiocarcinoma (pCCA) has been successfully treated using strict patient selection criteria combined with neoadjuvant therapy, achieving 5-year OS rates of 50–68%, though emerging data suggests chemotherapy-based approaches may be preferable to radiation in selected cases. Intrahepatic cholangiocarcinoma (iCCA), previously considered a contraindication to transplantation, can now achieve excellent long-term outcomes (79.5% 5-year OS) in patients demonstrating sustained response to neoadjuvant chemotherapy and radioembolization, with metabolic tumor volume < 70 cm^3^ serving as an objective prognostic marker. Across these three emerging indications, successful outcomes depend on strict patient selection based on tumor biology, intensive multimodal neoadjuvant therapy, multidisciplinary evaluation in high-volume centers, and careful observation during treatment to exclude patients with aggressive disease. This evolution in transplant practice offers curative intent therapy to patients that previously only had palliative therapeutic options, fundamentally transforming hepatobiliary and oncologic surgery.

## Simple Summary

Historically, liver transplantation was indicated for patients with cirrhosis and hepatocellular carcinoma. Over the last decade, carefully selected patients with colorectal liver metastases, perihilar cholangiocarcinoma, and intrahepatic cholangiocarcinoma have achieved long-term survival after liver transplantation that approaches outcomes of traditional indications. In this narrative review we discuss the key data including the Oslo SECA I–II series, the randomized TransMet trial for colorectal liver metastases, the Mayo Clinic protocol for perihilar cholangiocarcinoma, and prospective work from the Houston Methodist–MD Anderson collaborative group in intrahepatic cholangiocarcinoma into practical selection criteria, peri-transplant therapy strategies, and post liver transplantation management considerations to guide clinicians and researchers.

## 1. Introduction

Liver transplantation (LT) has long been recognized as the only potentially curative treatment for end-stage liver disease and, since the landmark Milan criteria were established in 1996, for patients with early-stage hepatocellular carcinoma (HCC) [1]. The Milan criteria—which limit transplantation to patients with a single tumor ≤ 5 cm or no more than three tumors each ≤3 cm—became the international gold standard for HCC selection and remain associated with 5-year OS rates between 60 and 80% and 5-year recurrence rates below 10% [2]. However, the application of transplantation to other primary and secondary hepatic malignancies has historically been cautious, with three major oncologic indications—colorectal cancer liver metastases (CRLM), perihilar cholangiocarcinoma (pCCA), and intrahepatic cholangiocarcinoma (iCCA)—long considered absolute or relative contraindications due to poor outcomes, high recurrence rates, and concerns regarding allocation of scarce donor organs [3].

The evolution toward transplant oncology has been driven by several critical insights. First, recognizing that poor outcomes were not inherent to transplantation itself but rather resulted from inappropriate patient selection, with early series including patients with extrahepatic metastases, vascular invasion, and advanced disease [1,4]. Second, the development of effective neoadjuvant chemotherapy regimens—particularly gemcitabine-cisplatin for cholangiocarcinoma (CCA) and modern multiagent regimens for colorectal cancer—created the opportunity to assess tumor biology through response to therapy [5,6]. This “test of time” principle, rooted in observations by Scheele and Wagner regarding colorectal disease progression on chemotherapy, allowed identification of patients with favorable tumor biology and those destined for rapid recurrence [7,8]. Third, advances in surgical technique, immunosuppression, perioperative management, and living donor transplantation improved overall transplant outcomes, making pursuit of novel oncologic indications increasingly feasible. Finally, recognition that the liver possesses unique immunobiological properties, including potential immune tolerance mechanisms and the capacity to control certain malignancies through transplant-related effects suggested that LT might confer advantages beyond simple surgical resection.

Machine perfusion represents a transformative technology in LT with the potential to expand the donor pool by enabling safe utilization of extended criteria donors and marginal grafts. Normothermic machine perfusion has demonstrated a reduction in organ discard rates from 13.3% to 3.5%, while enabling functional viability assessment through measurement of oxygen consumption and lactate clearance, with the RESTORE trial showing that 72.7% of declined livers could be successfully rescued after viability testing [9,10,11]. Hypothermic oxygenated machine perfusion demonstrates improved graft survival (HR 0.45) and reduced ischemic biliary complications in donation after circulatory death livers (OR 0.31) [9,11]. As transplant oncology continues to expand indications for LT including hepatocellular carcinoma, CCA, and colorectal liver metastases, several strategies can mitigate the impact on other waitlist patients: living donor liver transplantation (LDLT) offers a viable alternative that circumvents MELD-based allocation, with studies demonstrating superior long term outcomes in HCC patients (5-year OS 82% vs. 73% for deceased donor transplantation), while marginal grafts from extended criteria donors may safely expand the organ pool for oncologic patients without impairing survival or recurrence free survival [12,13]. Machine perfusion may also be one of the strategies to mitigate the impact on other patients on the waiting list.

## 2. Colorectal Liver Metastases

Colorectal cancer (CRC) is the third most common cancer and the second leading cause of cancer-related mortality in the USA [14]. Screening colonoscopy plays a pivotal role in the detection of earlier stages of CRC. However, 50% of the patients diagnosed with CRC will still develop metastatic disease, with liver being the most common metastatic site [15].

For CRLM that is not amenable to local therapy, systemic therapy is the standard of care that extends median OS to ~24–36 months, but 5-year OS in unresectable disease remains ~10–15% [16]. Currently, liver resection (LR) remains a treatment of choice for patients with technically resectable CRLM, achieving a 5- and 10-year OS of 44–50% and 24–33%, respectively; nevertheless, recurrence rates are high, and only 20% are cured [17]. First recurrence typically is intrahepatic only in 43%, extrahepatic only in 36% and combined intra- and extrahepatic in 21% [18]. LT for unresectable CRLM has emerged as a promising therapeutic option following decades of abandonment due to historically poor outcomes. Since the pioneering work from Oslo, Norway beginning in 2006, a growing body of evidence from prospective trials, registries, and single-center experiences has demonstrated that highly selected patients can achieve survival outcomes comparable to established transplant indications [19,20,21] (Table 1).

The foundation of modern LT for CRLM was established through the Oslo University Hospital’s Secondary Cancer (SECA) trial series. The SECA-I trial (2013) represented the first prospective study demonstrating feasibility of LT for this indication. This pilot study enrolled 21 patients with unresectable, liver-only colorectal metastases who underwent deceased donor LT. Despite a concerning disease-free survival (DFS) of 0% at 2 years, OS was remarkably 60% at 5 years. DFS at 1 year was about 35%. All patients who were followed beyond 11 months post-transplant experienced disease recurrence, eventually resulting in approximately 90% recurrence rate. The high recurrence rate was predominantly characterized by slowly growing pulmonary metastases that were amenable to curative resection, suggesting that recurrence patterns after transplantation differ fundamentally from those after liver resection [22].

Building on lessons learned from SECA-I, the SECA-II trial implemented more stringent selection criteria including at least 10% response to chemotherapy, a minimum 1-year interval between primary tumor diagnosis and transplant listing, and liver-only disease confirmed by PET-CT. The 15 patients in SECA-II achieved a 5-year OS of 83% and disease-free survival of 35%, representing a substantial improvement over SECA-I [23]. These patients had significantly lower tumor burden, smaller lesions, and lower carcinoembryonic antigen (CEA) levels compared to SECA-I participants. A sub study, SECA-II Arm D, evaluated 10 patients using extended criteria for donors, demonstrating that marginal grafts could be safely utilized for this indication [24].

The Oslo group subsequently published long-term outcomes from 61 transplanted patients, reporting a 5-year OS of 50% for all patients, with those having an Oslo Score of 0–2 achieving 63.5% survival compared to only 8.3% for scores 3–4. These data established the Oslo Score as a critical prognostic tool and validated that patient selection based on favorable tumor biology could yield outcomes comparable to conventional transplant indications [25,26].

In a recent publication from the Oslo group, they retrospectively compared 10 patients with recurrent, liver-only CRLM who underwent LT with 43 patients who underwent repeat LR. The primary endpoint was 5-year OS; secondary endpoints were DFS and survival after recurrence (SAR). Five-year OS was significantly higher with LT than LR—90% (95% CI, 55.5–99.7%) vs. 44% (95% CI, 29.1–60.1%); *p* = 0.013. Recurrence occurred in six of 10 LT patients (median SAR, 116.6 months; 95% CI, 42.8–190.4) and in 32 of 43 LR patients (median SAR, 26.1 months; 95% CI, 21.4–30.7; *p* = 0.026). These results indicate significantly improved long-term OS and SAR with LT compared with repeat LR in patients with liver-only recurrent CRLM after prior hepatectomy [27].

The TransMet trial represents a watershed moment in transplant oncology as the first multicenter, prospective, randomized controlled trial directly comparing LT plus chemotherapy versus chemotherapy alone for permanently unresectable CRLM. Conducted across 20 European centers in three countries, the trial enrolled 94 patients between 2016 and 2021 [28]. The selection process involved validation by an independent multidisciplinary expert committee, ensuring only truly unresectable cases with favorable biology were included. In the intent-to-treat analysis, the 5-year OS was 56.6% for the transplantation plus chemotherapy arm versus 12.6% for chemotherapy alone (*p* = 0.0003). The per-protocol analysis revealed even more impressive results: 73.3% 5-year survival for transplanted patients compared to 9% for chemotherapy alone. The median progression-free survival (PFS) was 17.4 months versus 6.4 months respectively. Notably, 19% of patients allocated to transplantation experienced disease progression while awaiting organs, highlighting the critical need for prioritized allocation. The trial demonstrated that 74% of transplanted patients who developed recurrence had lung metastases, many of which were amenable to further curative treatment [28].

The University of Toronto group established one of the first North American LDLT protocols for unresectable CRLM (NCT02864485). Their prospective clinical trial, initiated in October 2016, assessed 81 referred patients through February 2023, with seven receiving LDLT, 22 undergoing resection after downstaging, and 48 serving as controls. The LDLT group demonstrated superior recurrence-free survival compared to resection (1-year: 85.7% vs. 11.4%; 3-year: 68.6% vs. 11.4%, *p* = 0.012), though OS did not differ significantly. The median time from initial assessment to transplantation was 15.4 months, reflecting careful patient selection and donor evaluation [29].

The University of Rochester program reported the largest single-center North American experience with LDLT for CRLM, involving 23 patients representing 10–15% of evaluated patients who met selection criteria. The protocol restricted LT to patients with liver-only, radiologically unresectable CRLM who have undergone resection of the primary tumor, achieved sustained response or stable disease on modern systemic and/or liver-directed therapies, and demonstrate favorable tumor biology as reflected by low Oslo and Clinical Risk Scores and absence of extrahepatic disease on comprehensive staging, including PET imaging. The study demonstrated safety and feasibility with OS proportion of 100% and 91% at 1 and 3 years and a recurrence-free survival proportion of 100% and 40% at 1 and 3 years, respectively [30].

The Mayo Clinic Florida group evaluated the feasibility and outcomes of deceased donor LT in 12 patients with CRLM using strict selection criteria and advanced graft perfusion protocols. The median time from listing to transplant was 31 days (range: 3–115 days). Donor livers were marginal—most were donation after circulatory death—and all but two underwent machine perfusion. Post-transplant outcomes demonstrated a median hospital stay of 5 days with no cases of primary nonfunction, post-reperfusion syndrome, acute kidney injury, hepatic artery thrombosis, or ischemic cholangiopathy. At a median follow-up of 413 days, both patient and graft survival rates reached 100%. Disease recurrence occurred in three recipients (25%), with evidence of disease control in one following additional therapy. These findings corroborate European data, suggesting that DDLT for strictly selected CRLM patients is clinically effective, offering excellent short-term survival and manageable recurrence rates, provided that rapid organ access and advanced perfusion capabilities are available [31].

In their recent study assessing outcomes for adult patients who underwent LT for CRLM in North America between 2020 and 2025, the ARTx-Onc group included 94 patients across 14 centers, predominately male (65%), with a median age of 50 years. Most primary tumors originated in the rectosigmoid colon, and the majority had synchronous (80%) and node-positive (N1, 43%) disease at diagnosis. At the time of LT, CEA had decreased to a median of 3 ng/mL from 35 ng/mL at CRLM diagnosis, reflecting good tumor control. Genetic variants included KRAS in 29%, TP53 in 23%, and BRAF in 5%. The median Oslo score at LT was 1, with 12% having progressive disease despite therapy. Patients underwent intensive pre-LT therapy: all had chemotherapy (median duration 19 months), 40% underwent prior liver resection, 23% received hepatic artery infusion pumps, and various ablative therapies were common. Graft sources included living donors (67%), standard donation after brain death (17%), and extended criteria or donation after circulatory death (16%). Postoperative outcomes showed a high rate of complications (56% had Clavien–Dindo grade III or higher), but 1-, 3-, and 5-year OS rates were strikingly favorable at 97.6%, 77.6%, and 67.7%, respectively. Recurrence-free survival at the same intervals was 66.6%, 44.9%, and 44.9%, with most recurrences being pulmonary (31%) or hepatic (13%). Median survival after recurrence was nearly 2 years. At last follow-up (median 19 months), 87% of recipients were alive [32].

Rasschaert et al. recently presented the first multicenter outcomes from the Belgian national LT protocol for unresectable CRLM. The cohort of 29 patients, transplanted after a median of 13 months of chemotherapy, achieved a 2-year OS of 90.1% and recurrence-free survival of 54.8%. Although 11 recurrences were observed (predominantly pulmonary), the median OS post-recurrence was 26.5 months, indicating favorable biology. The Belgian protocol harmonizes selection across all six centers, mandating stable disease for 6 months and an 8-week chemotherapy-free “test of time” to exclude rapid progressors. With 28 MELD points granted to CRLM patients upon listing for LT, the program anticipates approximately 15 transplants annually [33].

The COLT trial (Colorectal cancer: improving Outcome with Liver Transplantation) is an ongoing multicenter, non-randomized, prospective parallel trial conducted across multiple Italian centers (NCT03803436). The study includes hyperselected patients with liver-limited unresectable CRLM who are RAS and BRAF wild-type with curatively resected primary colon cancer. Outcomes are prospectively compared 1:5 with matched cohorts from the TRIPLETE trial of triplet chemotherapy plus anti-EGFR antibodies. The trial predicts a 40% gain in 5-year OS (70% vs. 30%) for the transplant arm [34]. The COLT trial has contributed to developing the LITORALE protocol, a standardized patient selection system implemented in Italian transplant centers, which has demonstrated improved post-transplant outcomes [35].

**Table 1:** Published and ongoing studies for Liver transplant in CRLM.

Study	Design	Center(s)	Number of Patients	OS (5-Year)	DFS (5-Year)
SECA-I [22]	Prospective pilot	Oslo, Norway	21 (LT)	60%	0% (at 2 years)
SECA-II [23]	Prospective	Oslo, Norway	15 (LT)	83%	35%
SECA-II Arm D [24]	Prospective (extended criteria)	Oslo, Norway	10 (LT)	Not reported	Not reported
TransMet [28]	RCT	Europe (20 centers, 3 countries)	94 randomized (47 LT arm, 47 CT arm); 38 received LT	73% (per-protocol); 57% (ITT)	PFS 17.4 months
COLT [34]	Prospective	Italy (multicenter)	Ongoing comparison	Expected 70%	Secondary endpoint
SOULMATE [36]	RCT	Sweden (multi-center)	Ongoing	Not yet reported	Not yet reported
Rochester (LDLT) [30]	Prospective	University of Rochester, USA	10 (LDLT)	Not reached	Not reached
Toronto (LDLT) [29]	Prospective	University of Toronto, Canada	33 (7 LDLT, 22 resection, 48 controls)	90% (3-year for LDLT)	Not reported
Oslo Long-term Follow-up [25,26]	Long-term	Oslo, Norway	61 (LT)	50% (all patients); 63.5% (Oslo Score 0–2)	18.30%
Mayo Clinic [31]	Prospective	Mayo Clinic, USA	12	Not reached	Not reached
Belgian Liver Transplant Centers [33]	Prospective	Belgium	29	(2-year OS) 90.1%	Not reported
ARTx-Onc Registry [32]	Multicenter registry	USA/Canada (multicenter)	Multiple centers enrolling	Not yet reported	Not yet reported

OS, overall survival; DFS, disease-free survival; CRLM, colorectal liver metastases; LT, liver transplantation; LDLT, living donor liver transplant; RCT, randomized controlled trial; ITT, intention-to-treat.

The SOULMATE study (Swedish study Of Liver transplantation for isolated colorectal cancer liver Metastases not suitable for Operation or ablation compared to best established treatment) (NCT04161092), is a randomized controlled trial evaluating whether LT with extended criteria donor grafts increases OS compared to best established treatment. This study specifically examines the use of extended criteria donors, including donation after circulatory death donors, to minimize impact on the standard transplant waiting list. The use of extended donor criteria will make the results globally applicable, as most countries face organ shortage [36].

### 2.1. Selection Criteria and Prognostic Scoring Systems

Multiple scoring systems and selection criteria have been developed and validated to identify optimal transplant candidates.

The Oslo Score incorporates four negative predictive factors for OS; each assigned one point: maximal diameter of largest lesion > 5.5 cm; pre-transplant CEA > 80 μg/L; progressive disease on chemotherapy; and interval from diagnosis to transplant < 2 years. Patients with Oslo Score 0–2 demonstrated median OS of 151.6 months and 5-year survival of 75.4%, compared to 50.8 months and 39.7% respectively for scores 3–5. The Oslo Score has been validated across multiple studies and centers [25,26].

Originally developed for liver resection, the Fong Clinical Risk Score evaluates five factors: node-positive primary tumor, disease-free interval < 12 months, >1 tumor, size > 5 cm, and CEA > 200 ng/mL. In the transplant setting, patients with Fong scores 0–2 demonstrated significantly longer disease-free survival than those with scores 3–4. The score has been extensively validated and correlates more closely with outcome than tumor number alone (r^2^ = 0.92 vs. 0.80 for 5-year survival) [28,37].

Metabolic tumor volume (MTV) assessed by PET-CT represents total tumor metabolic activity. A cut-off of 70 cm^3^ has been validated, with MTV-low patients achieving median OS of 68 months. Several high-volume US centers have incorporated MTV thresholds into their selection criteria [25,38].

The International Hepato-Pancreato-Biliary Association (IHPBA) and Organ Procurement and Transplantation Network (OPTN) published consensus guidelines in 2021 providing a comprehensive framework for LT in unresectable CRLM. These guidelines standardize nomenclature and define management principles across five key domains: patient selection, evaluation of biological behavior, graft selection, recipient considerations, and outcomes. The guidelines recommend considering transplant only when metastases are unresectable by standard, complex, or combinatorial liver-directed approaches. Notably, current IHPBA guidelines do not exclude patients with right-sided primaries or KRAS mutations, though these factors carry prognostic significance [39]. The Organ Procurement and Transplantation Network guidelines define how candidates with liver tumors can be listed, prioritized, and re-prioritized through standardized and non-standard MELD exception pathways, so that tumor-related urgency (e.g., dropout risk) is incorporated into allocation when it is not captured by calculated MELD [40].

### 2.2. Key Prognostic Factors and Patient Selection

Primary tumor laterality significantly impacts outcomes. Right-sided primary tumors are associated with a substantially worse prognosis, with 5-year OS of 10% versus 60.1% for left-sided tumors (*p* < 0.001) in Oslo analysis. Some centers require extended observation periods (e.g., 18 months) for right-sided or KRAS-mutated tumors, achieving 3-year survival rates up to 91% with this approach [34,41]. CEA level ≤ 80 μg/L is associated with improved progression-free and OS across multiple studies. Tumor size with largest lesion ≤ 5.5 cm is incorporated into multiple scoring systems and represents an important selection criterion [26,42]. R0 resection of the primary tumor is required in virtually all protocols. Primary tumor T-stage ≤ T3 and N-stage ≤ N2 are preferred in some protocols. Nodal status of the primary tumor carries prognostic significance, with higher N-stage associated with worse outcomes [42].

BRAF mutation status is considered an exclusion criterion in most contemporary protocols including TransMet. RAS mutation status influences prognosis, with KRAS wild-type patients preferred, though KRAS alteration is not universally excluded [43,44]. A recent molecular analysis revealed that KRAS/TP53 co-mutations were significantly associated with worse outcomes [45]. Microsatellite instability (MSI-high) and mismatch repair deficiency (dMMR) are considered exclusion criteria in current protocols as these tumors are potentially curable with immune checkpoint inhibition (ICI) and use of ICI is a contraindication in a post-transplant recurrent setting [46,47,48].

Response to pre-transplant chemotherapy is universally recognized as a key predictor of long-term outcomes. The SECA-II protocol required at least 10% response to chemotherapy, while other protocols specify stable or responding disease. Duration of pre-transplant chemotherapy ranges from 6 to 12 months in most protocols, allowing adequate assessment of tumor biology while minimizing treatment-related toxicity [23,42].

While post-transplant adjuvant chemotherapy for CRLM is not mandatory, it is commonly recommended and left to the discretion of the treating oncologist. In the TransMet trial, post-transplant chemotherapy was recommended but not required, with 68% of transplanted patients ultimately receiving it; the combination of chemotherapy with immunosuppression showed acceptable toxicity (36% grade 3–4), though no clear clinical benefit could be demonstrated due to limited patient numbers [28]. In the SECA I and II experience, chemotherapy was primarily administered at recurrence rather than as routine adjuvant therapy [22,23]. Current practice favors an individualized, multidisciplinary approach balancing potential oncologic benefit against the risks of combining cytotoxic therapy with immunosuppression.

Role of circulating tumor DNA (ctDNA) monitoring before LT is not yet established but it may be an emerging tool for patient selection, prognostication and surveillance. In a small series of five liver transplant recipients for CRLM, three of four patients with positive pre-transplant ctDNA achieved ctDNA clearance post-transplant and remained disease-free, suggesting that pre-transplant ctDNA positivity alone may not preclude successful transplantation [49]. Post-transplant ctDNA monitoring appears more informative for predicting recurrence. Patients with detectable post-transplant ctDNA experienced recurrence at higher rates (50%) compared to those who were ctDNA-negative (25%), though this difference did not reach statistical significance in small cohorts. This may be a high-risk subgroup in which “adjuvant” chemotherapy can be considered post-transplant. Further research and studies are needed on the utility of chemotherapy in this setting as LT demonstrated the capacity to clear ctDNA in approximately 50% of patients with sequential testing, with clearance patterns of ±(pre-transplant positive to post-transplant negative) observed in half of monitored patients [50]. Ongoing research is evaluating ctDNA’s utility for patient selection, surveillance protocols, and as an indication for treatment intensification or modification [51].

### 2.3. Recurrence Patterns and Post-Transplant Management

In patients undergoing LT for CRLM, the lungs represent the predominant site of recurrence, with pulmonary metastases most often appearing as single-site lesions that demonstrate indolent growth. These pulmonary recurrences exhibit unique biology, as some nodules may be detected prior to LT without adversely impacting survival, and they frequently remain amenable to local treatment. In contrast, hepatic recurrence as a solitary first site is exceedingly rare after LT and typically occurs only within the context of multifocal metastatic disease. This pattern contrasts sharply with recurrence following liver resection, where liver-only metastases constitute the most common initial relapse site. Patients developing metastases within the transplanted liver tend to have worse prognoses, reflecting advanced disease dissemination [52,53].

Approximately half of patients who undergo LT for CRLM eventually experience disease recurrence, with the lung being involved in nearly half of those cases. Despite this high recurrence rate, 5-year OS post-transplant remains around 50%, likely reflecting the aggressive management of pulmonary lesions and the biological implications of removing the native liver harboring the primary metastases [52,53].

The treatment of recurrent disease following LT for CRLM typically prioritizes aggressive, curative-intent management, especially for pulmonary recurrences, which are most common. Surgical resection of pulmonary metastases is frequently feasible and associated with excellent long-term outcomes; in selected patients, 5-year OS after pulmonary metastasectomy can reach 70%, and in those with highly favorable risk profiles, 10-year survival after resection has been reported as high as 76–100% [26]. This outcome appears notably superior compared to conventional management of relapses after hepatic resection. Close surveillance to identify resectable metastatic lesions early, coupled with an active surgical approach, is recommended for maximizing survival after transplant. In cases of disseminated or unresectable recurrence, systemic chemotherapies therapies may be considered, following similar principles as for advanced colorectal cancer in non-transplant patients [26,54].

In instances of disease recurrence after LT, immunosuppression is commonly adjusted to reduce overall oncologic immunosuppressive pressure while maintaining graft safety, with consensus guidance favoring incorporation of an mTOR inhibitor into the maintenance regimen. The IHPBA consensus guideline on LT for non-resectable CRLM describes a panel-recommended approach of either gradually tapering a calcineurin inhibitor (CNI) while adding an mTOR inhibitor or initially using a CNI and then converting to an mTOR inhibitor after approximately 4–6 weeks to mitigate the wound-healing complications associated with early mTOR-inhibitor exposure. In parallel, the guideline framework supports minimizing CNI exposure when feasible and transitioning toward an mTOR-based platform as part of post-transplant oncologic management, recognizing that these recommendations are largely consensus-driven in an evolving evidence base [39].

## 3. Cholangiocarcinomas

CCAs collectively represent one of the most aggressive hepatobiliary malignancies, accounting for the second leading cause of primary liver cancer after HCC. Both pCCA and iCAA subtypes carry exceptionally poor prognoses, with 5-year OS rates for all stages and subtypes historically reported at less than 10% regardless of treatment modality. pCCA, also known as Klatskin tumor, comprises more than 50% of all CCA cases and typically presents at advanced stages due to its central location at the hepatic hilum and the insidious nature of early biliary obstruction. Traditional surgical management of pCCA, despite aggressive hepatectomy combined with en bloc vascular resection and caudate lobe resection, has yielded median OS of only 16–30 months with postoperative mortality rates of 10–15% and 5-year survival rates of 25% [4,55,56]. For iCCA, the situation is even more challenging: 70–80% of patients present with unresectable disease due to advanced T stage, vascular invasion, or extent of liver involvement. Surgical resection, when feasible in the 20–30% of patients with resectable disease, is associated with postoperative recurrence rates of 50–60% and 5-year survival of only 25–40% [57,58].

ctDNA has emerged as a valuable tool in CCA management, serving multiple clinical roles including molecular profiling, treatment monitoring, and prognostic assessment [59]. ctDNA testing successfully identifies actionable molecular alterations in approximately 18% of patients, including IDH1 mutations (11%), FGFR2 fusions (9%), BRAF V600E mutations, and HER2 amplifications, with performance comparable to tissue-based testing for hotspot mutations though with lower sensitivity for certain gene fusions [59]. Beyond initial genotyping, ctDNA demonstrates significant utility for monitoring treatment response and detecting resistance mechanisms, with studies showing that 63% of treatment-naïve patients had mutational profile changes during chemotherapy and 24% of patients on targeted therapies developed emergent RAS alterations at progression [60,61]. In the liver transplant setting specifically, ctDNA shows promise for both pre-transplant risk stratification and post-transplant surveillance, with proof-of-concept data demonstrating that patients with post-transplant ctDNA positivity experienced recurrence at higher rates (50% vs. 25% in ctDNA-negative patients), and that LT can achieve ctDNA clearance in 50% of patients with sequential testing, with median tumor mutation burden decreasing from 1.23 mut/Mb pre-transplant to 0.00 mut/Mb post-transplant [50,62]. Serial ctDNA monitoring enables real-time assessment of treatment efficacy, early detection of molecular resistance, and in the post-surgical/transplant setting, can detect recurrence with 93.8% sensitivity and an average lead time of 3.7 months compared to standard imaging surveillance, positioning it as an increasingly important complement to tissue-based molecular testing and traditional tumor markers in CCA care [63,64].

### 3.1. Perihilar Cholangiocarcinoma

Initial efforts investigating LT for CCA reported suboptimal results with recurrence rates of around 50% and 5-year survival rates between 23 and 42% [65,66]. This initially led to the procedure being deemed contraindicated [67].

The Mayo Clinic established a comprehensive protocol for LT for unresectable pCCA initially described in 2000 [68] and subsequently updated by Heimbach et al. in 2004 [69]. The series included 56 patients with stage I and II pCCA treated with neo-adjuvant external beam radiation and trans-catheter brachytherapy in combination with fluoropyrimidine-based systemic therapy prior to planned LT [69]. The reported 5-year survival among patients undergoing LT was 82%, comparable to outcomes from other accepted LT indications. This led to acceptance of unresectable pCCA as a standard of care indication for carefully selected patients. Subsequent publications including data from 349 patients enrolled for treatment with the neoadjuvant protocol between 1993 and 2018 show similar results with a 5-year and 10-year survival of 69% and 62% after transplantation.

Notably, the dropout rate from complications, progressive disease or extrahepatic disease found on staging is between 25 and 40% [70,71], leading to 5-year and 10-year survival of around 51% and 46% amongst all who start neoadjuvant therapy. Multi institutional data has demonstrated similar findings [70,72].

A distinct difference has been noted between pCCA arising in the setting of primary sclerosing cholangitis (PSC) and pCCA arising de novo, without this underlying disease. PSC is a well-established risk factor for CCA, with between 7 and 11% of these patients developing CCA [73,74,75,76]. In the setting of pCCA arising from PSC, the 5- and 10-year survival post-LT is 76% versus 58% for de novo pCCA [71]. For comparison, in carefully selected patients that were able to undergo resection, the 5-year survival rates are between 35 and 44% [77,78]. Given the favorable outcomes in patients with PSC and CCA combined with the fact that transplantation may be effective treatment of their underlying liver disease and greatest oncological risk factor for subsequent liver malignancies, transplantation is advocated as a preferred curative treatment strategy in these patients [71] with MELD exception points given [79]. Currently, CCA is still a fairly uncommon indication for LT with less than 100 cases/year performed in the United States. There is interest in expanding indications beyond unresectable de novo pCCA and pCCA in the setting of PSC.

Whether transplantation should be the preferred curative treatment modality for patients with de novo resectable hilar CCA remains an area of debate. Crome et al. published a retrospective study comparing patients treated at the Mayo Clinic who underwent resection (n = 99) to patients undergoing transplantation protocol (n = 54) [80]. They found that progression-free survival (PFS) and OS were improved in the LT group compared to the resection group: 5-year PFS 54% versus 29%; 5-year OS 59% versus 36%. However, a subgroup analysis including patients with no lymph node metastases and that achieved negative margins of resection (admittedly something which is unknown preoperatively and therefore cannot be used as a selection criteria) failed to demonstrate the superiority of transplantation over resection. Those with more “borderline resectable” tumors with extension into bilobar segmental intrahepatic ducts necessitating multiple biliary anastomoses for reconstruction did benefit from transplantation.

Conversely, Ethun et al. demonstrated that even in those patients with de novo pCCA with tumors < 3 cm and node-negative disease treated within US Extrahepatic Biliary Malignancy Consortium, survival was better with transplantation protocol (n = 35) rather than resection (n = 57) (41% versus 27% 5-year survival, *p* = 0.049) in the intention-to-treat analysis [81]. While this may argue that the benefit of transplantation extends to these patients and listing criteria should be expanded, others have argued that the difference of 14% in long-term survival is not enough to justify allocation of scarce organs for such an indication. Furthermore, the survival in the resection group in this retrospective study is lower than in other series groups [71].

The TRANSPHIL trial (NCT02232932), a French prospective open-label randomized multicenter comparative study ongoing since 2012 aimed to clarify this debate about best treatment for resectable de novo pCCA. Initiation of either approach (transplant or resection) precludes switching to the other. Violation of the surgical plane in incomplete resection is generally a contraindication to transplantation as these patients have not traditionally done well with subsequent transplantation. Additionally, neoadjuvant ablative chemoradiation may induce inflammation, fibrosis, and late treatment field toxicity, which can increase technical complexity of subsequent surgery in the irradiated hilum or liver parenchyma [82]. The study was terminated early, probably due to the high dropout rate among randomized patients randomized to the transplant arm (55%) in addition to slower rate of enrollment [83].

### 3.2. Intrahepatic Cholangiocarcinoma

iCCA has historically been viewed as a contraindication for transplantation. Early studies indicated very poor outcomes, with high rates of recurrence and elevated short-term mortality [84,85].

More contemporary data suggests that very early iCCA may have a distinct, more favorable biology that is compatible with acceptable post-transplant outcomes. Several retrospective series evaluating patients transplanted for presumed HCC or decompensated cirrhosis who were found to have iCCA on explant have shown encouraging results when disease is limited to a single lesion ≤ 2 cm without macrovascular invasion. In a multicenter study of 15 such patients, 1-, 3-, and 5-year survival was 93%, 84%, and 65%, with recurrence rates of 7%, 18%, and 18%, respectively [86]. When pooled with additional cohorts meeting very-early criteria, the estimated recurrence risk is approximately 9%, supporting reconsideration of transplantation in this narrowly defined subgroup [86,87,88].

Another study assessed outcomes after LT in patients transplanted for presumed HCC who were subsequently found on explant to have iCCA or combined HCC–cholangiocarcinoma. Recurrence was more frequent in the isolated iCCA (n = 17) and mixed HCC–iCCA (n = 27) groups than in the HCC cohort (n = 574), with rates of 36.4% versus 10.8%. For cases deemed “very early” iCCA on preoperative imaging (single lesion ≤ 2 cm without vascular invasion), 1- and 5-year survival were both 63.6% and recurrence was 33.3%, significantly higher than the 11% recurrence observed in patients within the Milan criteria for HCC (*p* = 0.02), while 5-year survival remained similar (70.3% in the HCC cohort). Pretransplant imaging appeared to under-stage iCCA in two-thirds of patients (66.7%) [89].

In a study published by the French group, they evaluated outcomes in patients with iCCA or mixed CCA–HCC with the largest tumor nodule ≤ 5 cm on explant after LT performed for cirrhosis or HCC (n = 49) and compared them with patients undergoing resection for iCCA meeting the same tumor-size criteria. After a median 25-month follow-up, 1-, 3-, and 5-year OS was 90%, 76%, and 67% after transplantation versus 92%, 59%, and 40% after resection (*p* = 0.17). Recurrence was significantly less frequent after LT (27% vs. 58%, *p* = 0.008), and recurrence did not differ between tumors < 2 cm and those 2–5 cm, unlike prior reports. Given the reported 82% survival for resectable iCCA < 2 cm, the authors note surgery may remain preferred for resectable early-stage disease, while these data support considering LT for iCCA < 5 cm arising in cirrhosis or for tumors < 2 cm not amenable to resection, warranting prospective trials in this setting [90].

The Methodist–MD Anderson Joint Cholangiocarcinoma Collaborative Committee established a prospective protocol in 2010 for patients with locally advanced, unresectable iCCA deemed ineligible for curative resection by multidisciplinary review. The protocol intentionally abandons simple size-based selection and instead uses chemosensitivity and radiographic stability as the primary gatekeepers for LT candidacy [91]. Key selection principles include histologically confirmed iCCA; AJCC stage I–II without extrahepatic disease or macrovascular invasion; unresectable but liver-limited disease; and ≥6 months of objective radiographic response or stability on systemic therapy before listing; this waiting period functions as an in vivo stress test of tumor biology, allowing time for biologically aggressive tumors to declare themselves through progression or dissemination. Mandatory operative staging with exploration, regional lymphadenectomy, and peritoneal assessment is required prior to LT to exclude occult nodal or peritoneal metastases, given the limited sensitivity of imaging for micro metastatic spread [92]. The protocol is built around modern biliary tract cancer systemic therapy, most commonly gemcitabine–cisplatin (GemCis) based on the ABC-02 trial, which established GemCis as the first-line standard for advanced biliary tract cancer including iCCA [93].

The initial prospective case series from this program reported six transplanted patients with locally advanced, unresectable iCCA who had responded to neoadjuvant chemotherapy. Reported OS after LT was 100% at 1 year and 83% at both 3 and 5 years, with recurrence-free survival of 50% at 1, 3, and 5 years; median time to recurrence among those who relapsed was about 8 months. Notably, donor organs included extended-criteria and domino grafts, underscoring the feasibility of using marginal grafts in highly selected oncology candidates [91]. An expanded cohort with longer follow-up included 18 transplanted patients among 32 listed between 2010 and 2021 [92]. In this series, OS after LT was approximately 100% at 1 year, 71% at 3 years, and 57% at 5 years, despite a median cumulative tumor diameter > 10 cm and a median of 2 lesions, reflecting genuine “locally advanced” disease. Recurrence occurred in ~39% of transplanted patients, but non-transplanted listed patients had extremely poor outcomes, with essentially no 2-year survivors, highlighting the potential absolute survival gain from LT in this setting [92].

These outcomes strongly support the central premise that tumor biology, not raw tumor burden, is the key determinant of post-LT results in iCCA. Patients with large or multifocal tumors who show durable response or stability on neoadjuvant therapy can achieve survival markedly better than historical unselected iCCA LT cohorts. Conversely, failure to respond to systemic therapy is treated as a negative selection marker, and such patients are typically excluded from LT.

Based on the data from the Methodist–MD Anderson Joint Cholangiocarcinoma Collaborative Committee and the French group (Table 2), the Organ Procurement and Transplantation Network has approved criteria for LT for iCCA. In general, the criteria are more restrictive than those outlined by the Methodist–MD Anderson Joint Cholangiocarcinoma Collaborative Committee [92]. Continued future directions should involve efforts to expand criteria with preservation of outcomes seen in more limited disease. Patient selection may be improved by better imaging modalities and possibly tumor-informed circulating tumor DNA assays which have recently demonstrated prognostic value in iCAA [94]. Similar to pCCA, proceeding with a LT protocol may allow for delivery of ablative doses of treatment that would otherwise limit viability of the native liver. As systemic therapy options improve, targeted therapies with superior response rates may be moved to the neoadjuvant setting. Already, since the time of the McMillan publication, immunotherapy has been incorporated as part of frontline therapy. Previous studies focused on HCC have shown the pre-LT use of immunotherapy to be safe pending a washout period of about 3 months [95,96].

## 4. Conclusions

LT has evolved from therapy exclusively for cirrhosis and hepatocellular carcinoma into a curative option for carefully selected patients with unresectable CRLM, pCCA, and iCCA. This paradigm shift reflects recognition that poor historical outcomes reflected inappropriate patient selection rather than inherent transplant failure. Across all three indications, rigorous biology-based selection criteria—emphasizing response to neoadjuvant therapy, absence of progressive disease, and extrahepatic metastases—identify patients achieving long-term cure: CRLM achieves 73.3% five-year OS compared to 9% with chemotherapy alone; pCCA achieves 71–74% 5-years post-transplant survival; and iCCA achieves 57–83% 5-year OS, dramatically exceeding historical unselected iCCA controls of 18–25%.

Success requires strict selection, intensive multimodal neoadjuvant therapy, multidisciplinary evaluation in high-volume centers, and careful surveillance to exclude progressive disease. Post-transplant recurrence (30–50%), vascular complications (20–40% in pCCA), and dropout during neoadjuvant therapy (25–40%) remain significant challenges requiring enhanced imaging, molecular biomarkers, and refined therapeutic strategies. Integration of circulating tumor DNA testing, PET-CT, and modern systemic agents into neoadjuvant protocols warrants prospective investigation to maintain oncologic efficacy while reducing toxicity. Standardization through multi-center trials and collaborative registries will establish transplant oncology as an evidence-based discipline with reproducible outcomes. As transplant oncology matures, cautious expansion to other hepatic malignancies should proceed through rigorous pilot protocols with careful attention to organ allocation ethics and long-term outcome validation.

## 5. Future Directions

Advancing transplant oncology depends on leveraging circulating tumor DNA and molecular profiling (including KRAS/TP53 assessment for CRLM and FGFR/IDH testing for iCCA) alongside standardized PET-CT imaging to refine patient selection; conducting prospective trials to evaluate chemotherapy only neoadjuvant strategies for pCCA as alternatives to radiation while exploring integration of targeted and immunologic therapies; establishing multicenter registries and standardized protocols to generate reproducible, evidence-based outcomes across high-volume centers; and developing biomarker-driven post-transplant surveillance systems coupled with investigation of adjuvant molecular targeted and systemic therapies tailored to individual tumor characteristics.

## Figures and Tables

**Table 2:** Proposed criteria for the transplantation of intrahepatic cholangiocarcinoma from the Organ Procurement and Transplantation Network compared to reported or currently recruiting clinical trials.

Criterion	OPTN Criteria (2024–2025) [40]	Houston Methodist/MD Anderson (NCT—Phase II) [92]	TESLA Trial (NCT04556214)	LIRICA (NCT06098547)
Tumor Size	≤3 cm	Locally advanced (no size limit)	No size limit (median 11.5 cm)	Not specified
Tumor Number	Solitary	Not specified	Multiple allowed (median 7 tumors)	Not specified
Histologic Confirmation	Required (biopsy-proven iCCA or mixed HCC-iCCA)	Required (histologically confirmed)	Required (histologically verified)	Required (histologically confirmed)
Cirrhosis	Required	Not required	Not required	Not required
Vascular Invasion	Not allowed	Not allowed	No major vascular invasion; intrahepatic only	Not allowed
Extrahepatic Disease	Not allowed	Not allowed	Not allowed	Not allowed
Lymph Node Status	Not specified	Not allowed	Not allowed (on imaging)	Not allowed
Treatment Duration	≥6 months	≥6 months	≥6 months	≥6 months
Disease Stability	Required (no growth, no new lesions)	Required (stability or regression on neoadjuvant therapy)	Required (response to neoadjuvant therapy)	Required (stability or regression)
Tumor Differentiation	Not specified	Not specified	Not specified	Not specified
CA 19-9 Level	Not specified	Not specified	Reported (median 198 kU/L)	Not specified
Performance Status	Not specified	ECOG ≤ 1	ECOG 0–1	ECOG 0–1

OPTN, Organ Procurement and Transplantation Network; iCCA, intrahepatic cholangiocarcinoma; ECOG, Eastern Cooperative Oncology Group; CA 19-9, Carcinoembryonic antigen 19-9.

## Data Availability

The original contributions presented in this study are included in the article. Further inquiries can be directed to the corresponding author.
